# Reducing motor evoked potential amplitude variability through normalization

**DOI:** 10.3389/fpsyt.2024.1279072

**Published:** 2024-01-31

**Authors:** Francisco Faro Viana, Gonçalo Cotovio, Daniel Rodrigues da Silva, Carolina Seybert, Patrícia Pereira, Artur Silva, Filipe Carvalho, Albino J. Oliveira-Maia

**Affiliations:** ^1^Champalimaud Research, Champalimaud Foundation, Lisbon, Portugal; ^2^Champalimaud Clinical Centre, Champalimaud Foundation, Lisbon, Portugal; ^3^NOVA Medical School, Faculdade de Ciências Médicas, NMS, FCM, Universidade NOVA de Lisboa, Lisbon, Portugal; ^4^Department of Psychiatry and Mental Health, Centro Hospitalar de Lisboa Ocidental, Lisbon, Portugal; ^5^Portuguese Red Cross Health School, Lisbon, Portugal

**Keywords:** transcranial magnetic stimulation, cortical excitability, motor evoked potential, normalization, variability

## Abstract

**Background:**

Transcranial Magnetic Stimulation (TMS) is used for *in vivo* assessment of human motor cortical excitability, with application of TMS pulses over the motor cortex resulting in muscle responses that can be recorded with electromyography (EMG) as Motor Evoked Potentials (MEPs). These have been widely explored as potential biomarkers for neuropsychiatric disorders but methodological heterogeneity in acquisition, and inherent high variability, have led to constraints in reproducibility. Normalization, consisting in scaling the signal of interest to a known and repeatable measurement, reduces variability and is standard practice for between-subject comparisons of EMG. The effect of normalization on variability of MEP amplitude has not yet been explored and was assessed here using several methods.

**Methods:**

Three maximal voluntary isometric contractions (MVICs) and 40 MEPs were collected from the right hand in healthy volunteers, with a retest session conducted 4 to 8 weeks later. MEP amplitude was normalized using either external references (MVICs) or internal references (extreme MEPs). Iterative re-sampling of 30 normalized MEPs per subject was repeated 5,000 times to define, for each normalization method, distributions for between-subject coefficients of variation (CV) of the mean MEP amplitude. Intra-class correlation coefficients (ICC) were used to assess the impact of normalization on test–retest stability of MEP amplitude measurements.

**Results:**

In the absence of normalization, MEPs collected from the right hand of 47 healthy volunteers were within reported values regarding between-subject variability (95% confidence intervals for the CV: [1.0567,1.0577]) and showed good temporal stability (ICC = 0.77). Internal reference normalization substantially reduced between-subject variability, by values of up to 64%, while external reference normalization had no impact or increased between-subject variability. Normalization with the smallest references reduced test–retest stability, with use of the largest references resulting in slight reduction or improvement of ICCs. Internal reference normalization using the largest MEPs was found to be robust to several sensitivity analyses.

**Conclusion:**

Internal, but not external, reference normalization reduces between-subject variability of MEP amplitude, and has a minimal impact on within-subject variability when conducted with the largest references. Additional research is necessary to further validate these normalization methods toward potential use of MEPs as biomarkers of neuropsychiatric disorders.

## 1 Introduction

Transcranial magnetic stimulation (TMS) is a non-invasive brain stimulation technique with several applications in the study of brain physiology. TMS stimulating devices, through coils of conductive material, generate a time-varying magnetic field perpendicular to the coil, that then induces electrical current in nearby conductive tissue. Anthony Barker and colleagues first showed in 1985, that application of TMS pulses over a specific spot of the motor cortex resulted in contractions of the abductor digiti minimi muscle of the contralateral hand, measured using surface electromyography (EMG), representing the first recordings of TMS-elicited motor evoked potentials (MEPs) (1). Changes in MEP peak-to-peak amplitude have been associated with dysfunction at several levels of the corticomotor pathway, allowing for insights into physiological characteristics of a variety of neurological and neuropsychiatric disorders, such as multiple sclerosis and depression (2–5). Additionally, medication has been shown to alter MEP amplitude (6–8) paving the way for use of this measure in drug development (9). While these and other findings are promising, the establishment of MEP amplitude as a research tool and biomarker has been contested due to conflicting evidence and lack of reproducibility (10–12) attributed, in part, to the high variability of MEP amplitude measurements. Indeed, MEPs have large trial-to-trial variability, thought to arise from rapid spontaneous fluctuations of corticospinal excitability (13, 14). Other factors such as TMS coil positioning methods are also known to impact MEP amplitude measurements (15, 16). Furthermore, sources of variability common to any technique dependent on surface EMG will also have an effect on these measurements. These include session-specific and subject-specific parameters such as electrode configuration and placement (17), the number of active motor neurons and the characteristics of the tissue between the surface of the muscle and the sensing electrodes (18).

In order to reduce the variability of MEP measurements, several authors have proposed that MEP amplitude estimation should be performed through averaging of multiple MEPs (19, 20). Others have suggested that the use of neuronavigation may increase our ability to revisit the same stimulation site and thus reduce the variability of the responses (15, 16). In EMG research, in addition to standardization of experimental procedures, e.g. skin preparation (21), a common practice to reduce the impact of session- and subject-specific parameters is normalization (22). Normalization consists in scaling the signal of interest to a known and repeatable value, usually extracted from the same muscle group, allowing for between- and within-subject comparisons across different sessions. A large number of EMG normalization methods have been proposed (23) and the most common method relies on representing the signal of interest as a ratio of the EMG amplitude to that resulting from maximum voluntary isometric contractions (MVICs). Another commonly applied method is using peak activation levels of the same muscle group obtained during the task under investigation (24). We anticipate that normalization may be a useful strategy to decrease between-subject variability of MEP amplitude and increase the stability of this measure across time. However, the effect of normalization on MEP amplitude variability has not yet been thoroughly explored. Here we aimed to study different normalization methods that can be of potential use in TMS-EMG, and understand their impact on MEP amplitude variability.

## 2 Materials and methods

### 2.1 Subjects

Here we analyzed MEP data collected in the context of a clinical study published previously (25). Briefly, participants were recruited at the Champalimaud Foundation, Lisbon, Portugal, and were eligible if there was no current diagnosis of a major neuropsychiatric disorder such as a mood disorder, substance use disorder, movement disorder, neuromuscular disorders or other uncontrolled medical conditions. Eligibility for TMS was assessed using a safety questionnaire adapted from previously published guidelines which included items regarding history of psychiatric and neurological disorder, loss of consciousness, hearing impairment as well as presence of metallic or magnetic implants, ongoing medication and pregnancy status (26). The study was conducted in accordance with the Declaration of Helsinki and was approved by the Champalimaud Foundation Ethics Committee. Written informed consent was obtained from all participants.

### 2.2 Experimental procedures

In this study, participants were asked to perform up to four TMS experimental sessions (25). In the first two sessions, MEPs were collected by applying TMS pulses over either the left or right motor cortex, in randomized order across visits. These sessions were performed with a two- to seven-day interval, to decrease the likelihood of potential carry-over effects. Four to 8 weeks later, these experimental sessions were repeated, in the same order as for the initial 2 sessions. In a second group of subjects, only two experimental sessions were performed with a 4 to 8 weeks interval, but with TMS pulses applied only over the left motor cortex. Experimental sessions were conducted in the afternoon, and for each participant, they were scheduled at the same time of day whenever possible. This was done to minimize potential effects of circadian rhythms on cortical excitability (27, 28). Data acquisition began with determination of the motor hotspot (M1) and resting motor threshold (RMT) in accordance with standard methodology. This was followed by the collection of MVICs from the right hand, independently of the hemisphere being assessed. Details on each of these methods are provided below.

#### 2.2.1 Electromyography recordings

**Figure 1:**
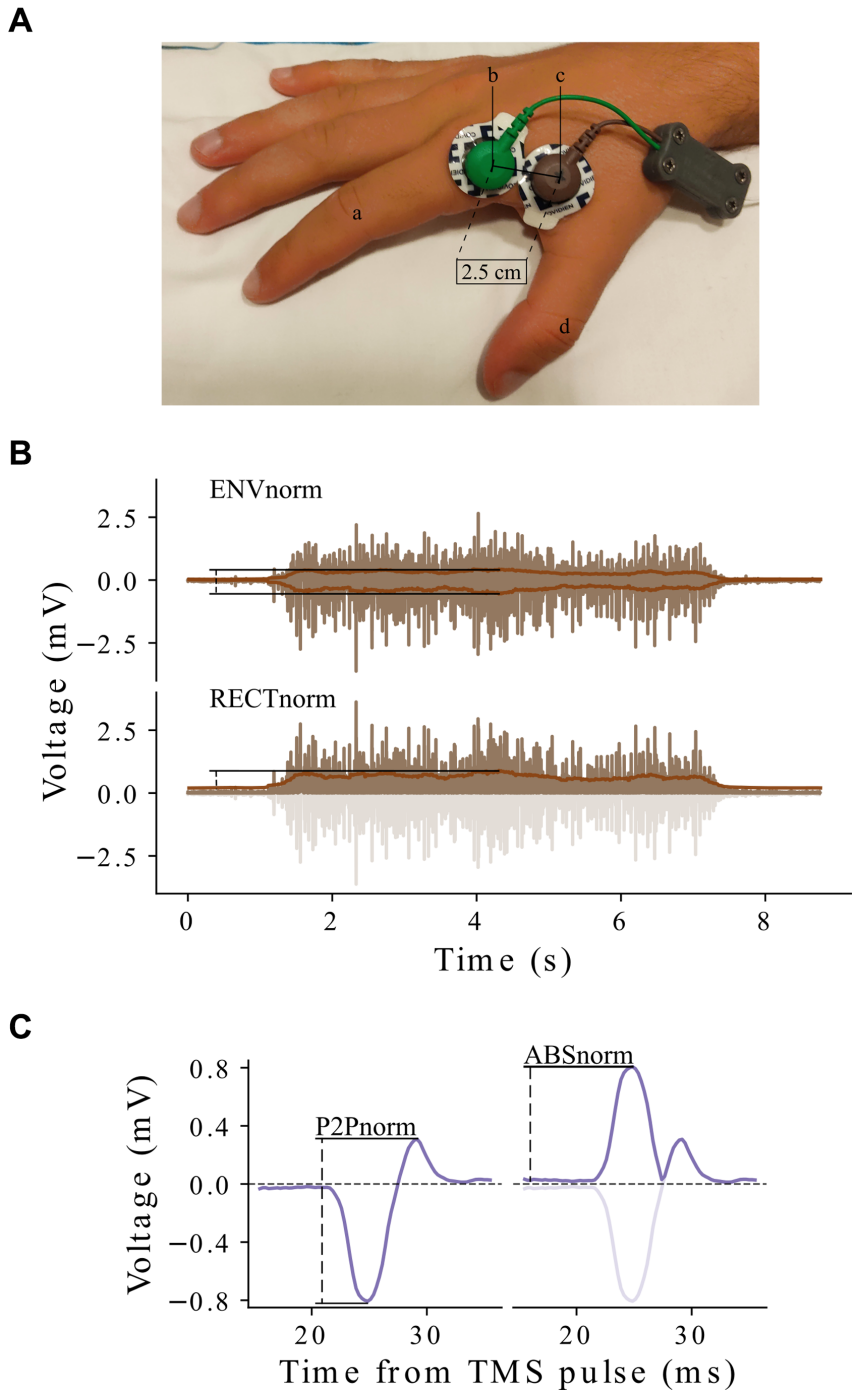
EMG electrode placement and normalization references. **(A)** An example of EMG electrode placement on one of the researchers’ hands: (a) index finger; (b) muscle tendon; (c) belly of the FDI; (d) thumb. One electrode is placed over the belly of the FDI while another electrode is placed distally with its center at approximately 2.5 cm in the direction of the muscle tendon. **(B)** External references: maximum MVIC amplitude after wave envelope (ENVnorm—top); maximum MVIC amplitude after wave rectification (RECTnorm—bottom); **(C)** Internal references: MEP peak-to-peak amplitude (P2Pnorm—left); MEP absolute maximum (ABSnorm—right). EMG, Electromyography; FDI, *first digiti interosseous*; MVIC, Maximum Voluntary Isometric Contraction; MEP, Motor Evoked Potential.

Electromyography was acquired with an in-house built acquisition system, at a sampling rate of 4,000 Hz and filtered analogically above 1 Hz and below 2000 Hz. This system was successfully validated against a commercial EMG acquisition system in a separate cohort (please see Supplementary material for details). In each session, participants were seated in a comfortable chair with both forearms at rest. In order to improve the quality of the electrode-skin interface, skin was cleaned and prepared using water sandpaper, followed by a quick wipe with an alcohol embedded gauze swab (21). Electromyography recordings were performed using medical grade Ag/AgCl based disposable cutaneous electrodes with a snap-on connector (Kendall^™^ H124SG). These electrodes already include a conductive and adhesive hydrogel, facilitating electrode placement procedures. Since EMG recordings are highly sensitive to changes in inter-electrode distance as well as electrode positioning, standardization of electrode placement across participants was achieved by placing one electrode over the belly of the *first digiti interosseous* (FDI) while another electrode was placed distally with its center at approximately 2.5 cm in the direction of the muscle tendon (Figure 1A). A ground electrode was placed over the left elbow to provide a zero-voltage reference point. To produce MVICs, researchers asked participants to make a ring shape by pushing the tip of their index and thumb against each other with maximum strength. After 3–5 s, participants were told to rest their fingers and relax for 1 min. Following the collection of three MVICs, TMS procedures were conducted.

#### 2.2.2 Transcranial magnetic stimulation

Transcranial Magnetic Stimulation procedures were performed under the recommended guidelines by the International Federation of Clinical Neurophysiology (20) using the MagPro X100 magnetic stimulator from MagVenture with a figure eight coil (Cool-B65). Motor hotspot for FDI was determined using a lycra swimming cap fitted on the head of participants, as described previously (29). Neuronavigation, Visor2^™^ software (ANT Neuro, Enschede, Netherlands), was used to aid coil positioning, improving the ability to precisely revisit the same stimulation site within sessions (15, 16, 30). Resting motor threshold was defined as the minimum intensity required to elicit MEPs of at least 50 μV in 5 out of 10 consecutive TMS pulses, as described previously (13). After defining the RMT, MEPs were collected by application of single TMS pulses over M1 at 120% of RMT. A total of 40 pulses were applied, ensuring a sufficient number of MEPs were collected to allow for reliable estimate of MEP amplitude per subject (31). A random inter-pulse interval of at least 6 s. was used to avoid stimulus anticipation (32). Additionally, pulse sequence was unpredictable to minimize target muscle pre-activation caused by anticipation of TMS pulses.

### 2.3 Data processing and analysis

#### 2.3.1 Preprocessing

Data processing and analysis was performed using custom-written scripts in Python3. Preprocessing began by applying a 3-level adaptive wavelet filter based on a Daubechies family wavelet (db1). Denoising threshold was calculated automatically using the Bayes Shrink algorithm (33). After wavelet filtering, signal smoothing was performed through the application of a third order Savitzky–Golay filter (34) with a window size of 5 samples. This denoising method was chosen as conventional filtering strategies produced signal distortions. Wavelet filtering is particularly useful in non-stationary signals and separating noise sources with overlapping frequencies (35–37). After signal denoising, a semi-automatic supervised algorithm was applied to segment the EMG trace into MVICs and MEP epochs and to determine their peak-to-peak amplitude. When MEPs presented a peak-to-peak amplitude smaller than 50 μV or if muscle pre-activation was identified, they were excluded from the analyses (38, 39).

#### 2.3.2 Normalization

Several normalization methods were tested to understand differential effects on MEP amplitude variability. For each acquisition session, normalization was performed with the signal references tested as normalization factors used to divide MEP peak-to-peak amplitude. Four reference signal types were tested following distinct approaches, two based on external references, and two based on internal references (see Figures 1B,C). External references were derived from MVICs. To obtain the first reference signal we applied a 200 msec moving average to the positive and negative portions of MVICs and drew an envelope around each contraction. We then calculated the envelope amplitude, defined as the peak-to-peak distance between maximum and minimum values - maximum MVIC amplitude after wave envelope (ENVnorm; Figure 1B—top). The other external reference signal was inspired by common EMG strategies to determine signal amplitude. First, the signal was rectified by taking the absolute value of the EMG trace; then, a 200 msec moving average was applied on the resulting signal to determine peak amplitude - maximum MVIC amplitude after wave rectification (RECnorm; Figure 1B—bottom). Internal references were based on MEP recordings. One was based on the peak-to-peak amplitude of MEPs (P2Pnorm; Figure 1C—left), while the other was based on MEPs’ absolute maximum—(ABSnorm; Figure 1C—right). To calculate the normalization factor for each method, we took the largest or smallest reference signals or the average of the two, or three, largest or smallest, reference signals (for MVICs, since only 3 were collected, the three largest and the three smallest MVICs are the same). Hence, we defined a total of 22 normalization methods (5 normalization factors × 2 external references +6 normalization factors × 2 internal references = 22 normalization methods) that were compared against the absence of normalization.

#### 2.3.3 Statistical analysis

The impact of normalization methods in between-subject variability was assessed using the coefficient of variation (CV). To allow for statistical comparison of CVs for the different normalization methods, a bootstrapping paradigm was applied as follows. First we performed iterative re-sampling of 30 normalized MEPs (nMEPs) per subject (5,000 iterations), with MEPs used for normalization factors excluded from the average when using internal references normalization methods. One participant was excluded from this analysis because less than 30 MEPs were available (31). Then, for each iteration, per subject nMEP amplitude was defined as the average of the randomly selected nMEPs. Finally, for each iteration, CV was calculated using the cohort mean and standard deviation of MEP amplitude across subjects. By performing these steps for each normalization method, a CV was calculated for each method 5,000 times, creating a distribution of CVs that was used to compare their performance. Since subjects repeated the MEP assessments 4 to 8 weeks after the first session, we also explored the test–retest stability of each method by calculating the intra-class correlation coefficients (ICCs) for absolute agreement using a two-way mixed-effects model (40).

The effect of the different normalization methods on MEP amplitude variability was first assessed on MEPs collected from the right hand resulting from stimulation of the left motor cortex. Motor evoked potentials collected from the left hand resulting from the application of TMS pulses over the right motor cortex were used to perform a confirmatory analysis of the effect of internal reference normalization methods on MEP amplitude variability.

## 3 Results

In the current study, 56 participants were identified from which 47 individuals were confirmed to be eligible (51.1% female, 36.7 ± 13.4 years old, Table 1). One participant did not perform the retest session. A more a detailed description of the study population can be found elsewhere (25).

**Table 1:** Socio-demographic characterization of the study population.

Total sample (N = 47)	N (%) unless stated otherwise
Age (mean ± standard deviation)	36.7 ± 13.4 years old
Female	24 (51.1%)
Higher education	40 (85.1%)
Handedness	
Self-report left hand dominance	4 (8.5%)
EHI (mean [range])	72.5 [−90, 100]
Coffee consumption	
none	15 (31.9%)
1 to 2 coffee’s a day	18 (38.3%)
3 or more coffee’s a day	14 (29.8%)
Chronic medication	22 (46.8%)

EHI, Edinburgh Handedness Inventory.

**Figure 2:**
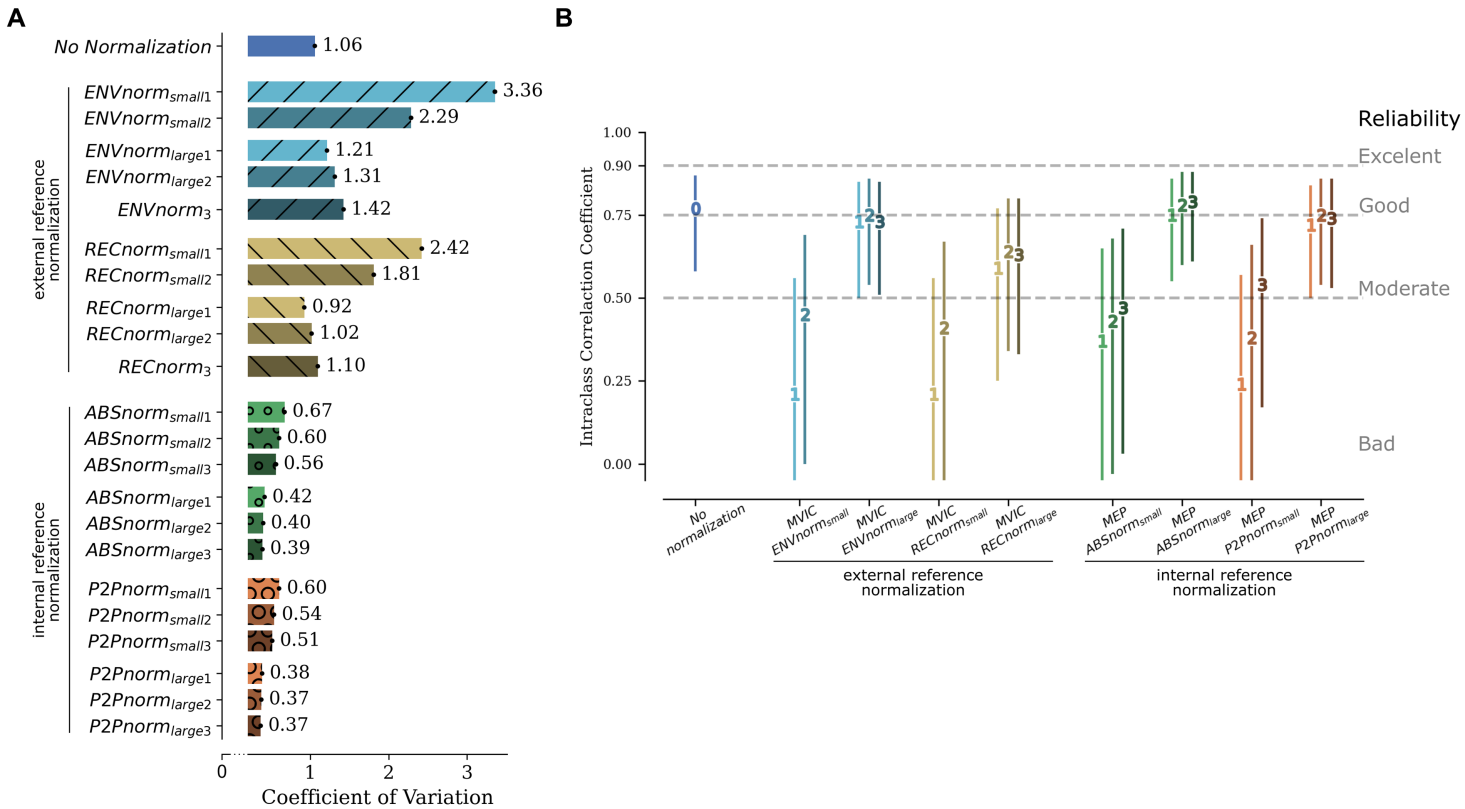
Variability for MEPs collected in the right hand. **(A)** Between-subject variability. Bars represent the mean CV for the 5,000 permutations, also represented as numbers next to the respective bar. The black line at the extremity of each bar represents the 95% Confidence Interval for the CV, that is very narrow given the high number of permutations. **(B)** Test–retest stability. Bars represent 95% Confidence Interval of the Intraclass Correlation Coefficient. Numbers on each bar represent the number of references used to calculate the normalization factor. ENVnorm, normalization based on maximum MVIC amplitude after wave envelope; RECnorm, normalization based on maximum MVIC amplitude after wave rectification; ABSnorm, normalization based on MEPs’ absolute maximum; P2Pnorm, normalization based on MEPs’ peak-to-peak amplitude; _small1_, _small2_, _small3_ and _large1_, _large2_, _large3_ refer to the use of the smallest, mean of 2 smallest, mean of 3 smallest, and largest, mean of 2 largest, mean of 3 largest references to calculate the normalization factor, respectively. Since only 3 MVICs were collected, the three largest and the three smallest ENVnorm or RECnorm references are the same; MVIC, Maximum Voluntary Isometric Contraction; CV, Coefficient of Variation; MEP, Motor Evoked Potential.

The effect of normalization on between-subject variability of MEPs collected in the right hand (i.e., stimulation of the left motor cortex) is represented in Figure 2A. In the absence of normalization, 95% confidence intervals (CI) of the CVs were [1.0567, 1.0577]. Using normalized MEPs, we found differences in between-subject variability between external and internal reference strategies. Specifically, with the exception of RECnorm when using the largest, or the mean of the two largest, references as the normalization factor, external reference methods increased between-subject variability. On the other hand, both internal reference normalization strategies resulted in a significant reduction of between-subject variability, particularly when using the 3 largest references to calculate the normalization factor, where the 95% CI of the CVs was [0.3886, 0.3892] when normalizing to ABSnorm_large3_ and [0.3653, 0.3660] when using P2Pnorm_large3_. This represents, across the two internal referencing methods a mean reduction of up to 64% in between-subject variability.

**Figure 3:**
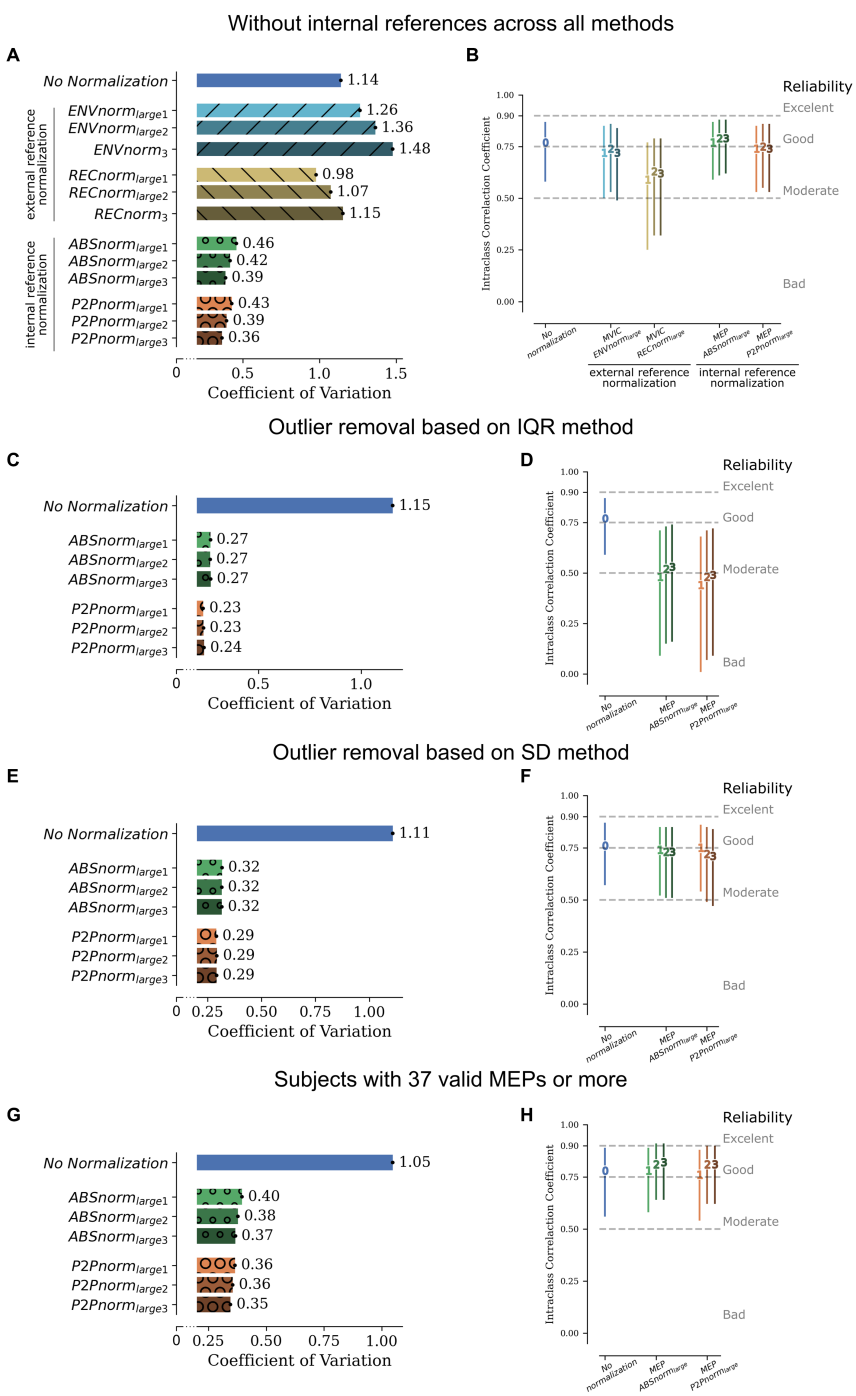
Sensitivity analyses for normalization methods based on largest references. Analyses were repeated after removal of potential sources of bias. **(A,B)** Removal of internal references across all normalization methods. **(C,D)** Pre-normalization removal of outliers based on the IQR method. (**E,F)** Pre-normalization removal of outliers based on the SD method. **(G,H)** Analyses restricted to subjects for whom at least 37 MEPs were available. Between-subject variability is represented in panels **A**,**C**,**E**,**G**. Bars represent the mean CV for the 5,000 permutations, also represented as numbers next to the respective bar. The black line at the extremity of each bar represents the 95% Confidence Interval for the CV, that is very narrow given the high number of permutations. Test–retest stability represented in panels **B**,**D**,**F**,**H**. Bars represent 95% Confidence Interval of the Intraclass Correlation Coefficient. Numbers on each bar represent the number of references used to calculate the normalization factor. ENVnorm, normalization based on maximum MVIC amplitude after wave envelope; RECnorm, normalization based on maximum MVIC amplitude after wave rectification; ABSnorm, normalization based on MEPs’ absolute maximum; P2Pnorm, normalization based on MEPs’ peak-to-peak amplitude; _small1_, _small2_, _small3_ and _large1_, _large2_, _large3_ refer to the use of the smallest, mean of 2 smallest, mean of 3 smallest, and largest, mean of 2 largest, mean of 3 largest references to calculate the normalization factor, respectively. Since only 3 MVICs were collected, the three largest and the three smallest ENVnorm or RECnorm references are the same; CV, Coefficient of Variation; IQR, Interquartile range; SD, standard deviation; MVIC, Maximum Voluntary Isometric Contraction; MEP, Motor Evoked Potential.

Regarding test–retest stability of right-hand mean MEP amplitude, calculated across all MEPs except those used for normalization (Figure 2B), in the absence of normalization, stability was good (ICC = 0.77, 95% CI [0.59, 0.87]). Normalization to the smallest, or mean of smallest reference signals, considerably reduced stability, while other normalization strategies had only a minimal impact. In fact, ABSnorm_large3_ normalization marginally improved MEP amplitude stability (ICC = 0.79, 95% CI [0.61, 0.88]). Due to its effect on test–retest stability, normalization to the smallest reference signals was thus not included in subsequent analyses. The apparent lack of impact of external references on between-subject variability also supported dropping these strategies in further analyses. However, since MEPs used as internal references are not included in the computation of mean normalized MEPs, the higher between-subject variability observed for external reference normalization methods could be due to the larger number of MEPs considered for these analyses. Thus, a sensitivity analysis was performed where all internal references were removed in all normalization approaches, including external reference normalization. The results of these sensitivity analyses were overlapping with those of initial analyses (Figures 3A,B), supporting the use of internal reference normalization methods, using the largest MEPs as references, for further analyses.

**Figure 4:**
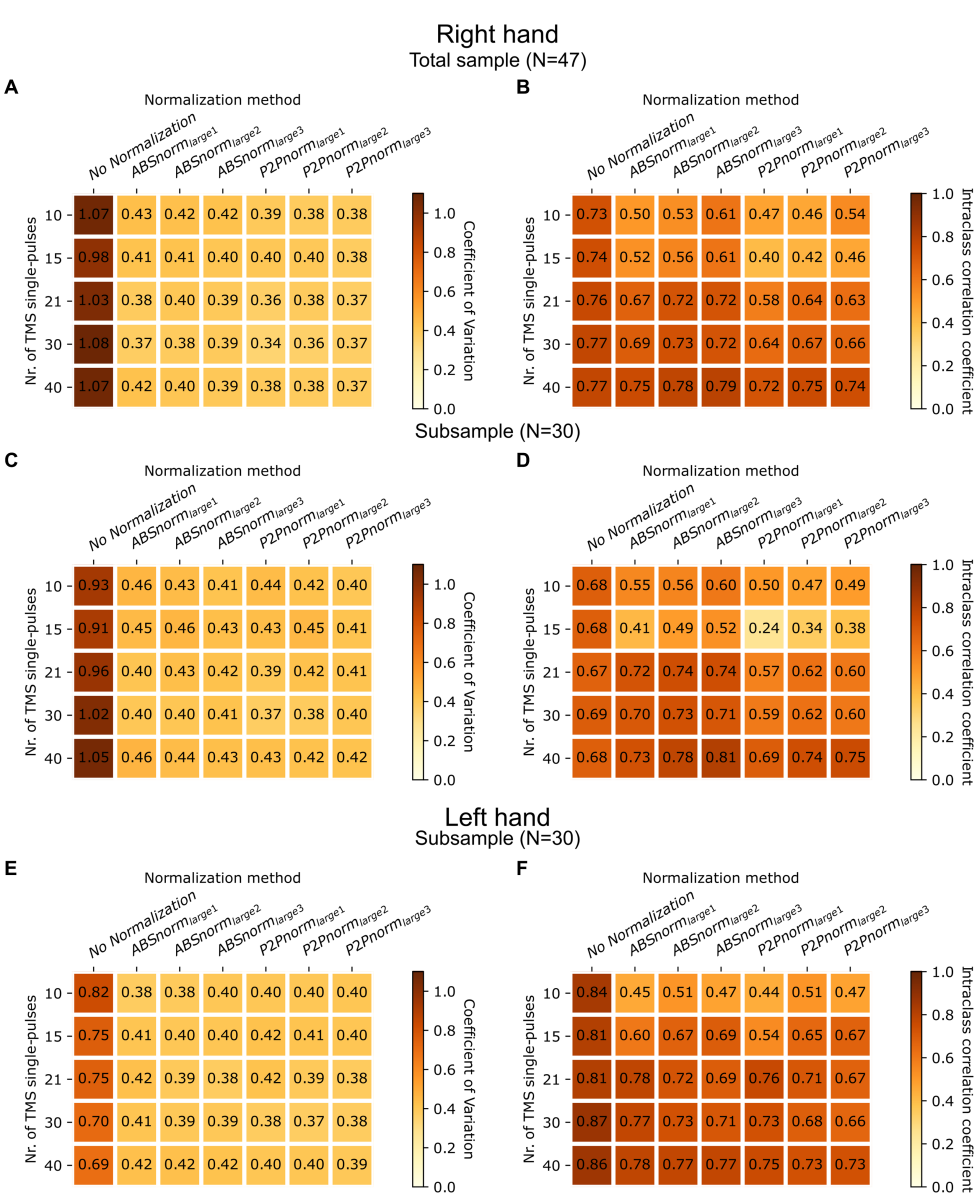
Generalizability and confirmatory analyses for normalization methods based on largest internal references. **(A,B)** Coefficients of variation (CVs) and Intraclass correlation coefficients (ICCs) were calculated for the different normalization procedures according to the first 10, 15, 21 or 30 MEPs collected in the right hand of each participant, and compared with calculations when using all MEPs collected (up to 40). **(C,D)** The analyses in **(A)** and **(B)** were repeated for right-hand MEPs in the subsample of subjects for whom MEPs were obtained both in the right and left hands. **(E,F)** CVs and ICCs according to the number of MEPs were also calculated for this subsample with data collected in the left hand. Between-subject variability is represented in panels **A**,**C**,**E**. Test–retest stability is represented in panels **B**,**D**,**F**. MEP, Motor Evoked Potential; ABSnorm, normalization based on MEPs’ absolute maximum; P2Pnorm, normalization based on MEPs’ peak-to-peak amplitude; _small1_, _small2_, _small3_ and _large1_, _large2_, _large3_ refer to the use of the smallest, mean of 2 smallest, mean of 3 smallest, and largest, mean of 2 largest, mean of 3 largest references to calculate the normalization factor, respectively.

In additional sensitivity analyses to assess robustness of internal reference normalization using the largest MEPs as references, we tested the impact of removing per-participant MEP outliers from analyses, before applying the normalization procedures. Two methods were used to detect outliers: the interquartile range (IQR) method, with removal of MEPs with an amplitude 1.5*IQR below the 1st quartile or above the 3rd quartile, or the standard deviation (SD) method, with removal of MEPs with an amplitude 2.5*SD above or below the mean MEP. In the absence of normalization, both methods of outlier removal led to an increase in between-subject variability (Figures 3C, 4E), when compared with analyses including all MEPs (Figure 2A). Test–retest stability did not seem to be compromised (Figures 3D,F) relative to original analyses (Figure 2B). When comparing nMEP variability to that obtained with the same method in the absence of outlier removal, the IQR method led to decreased between-subject variability (Figure 3C) but also less test–retest stability (Figure 3D). On the other hand, the removal of outliers based on the SD method led to a smaller decrease in between-subject variability in nMEP amplitude (Figure 3E), as well as a smaller impact on test–retest stability (Figure 3F). Finally, our bootstrapping analysis could be constrained by the fact that, particularly after removal of invalid MEPs, a relatively small number of MEPs could be available for some subjects. Analyses were thus repeated, restricted to data from subjects for whom at least 37 MEPs were available (n = 44), ensuring a large enough number of permutations was available to generate a distribution of CVs with 5,000 iterations. The results of these analyses (Figures 3G,H) were not qualitatively different from those performed previously (Figures 2A,B).

We also had concerns regarding the potential generalizability of MEP amplitude normalization using the largest MEPs as internal references. The number of TMS single-pulses applied to estimate mean MEP peak-to-peak amplitude per subject is quite variable across different research centers. To address the impact of collecting smaller numbers of MEPs, we separately calculated the CV and ICC for the first 10, 15, 21 and 30 MEPs collected for each participant, to compare with use of all MEPs (up to 40) for the same calculations. Bootstrapping was not performed as the aim of this analysis was to simulate real-world data collection of a smaller number of MEPs to calculate mean amplitude. Reducing the number of TMS pulses had negligible effects on between-subject variability, that followed the same pattern and values as the ones observed when using all of the available pulses (Figure 4A). Regarding test–retest stability, it is noteworthy that the number of TMS pulses did not impact ICCs when normalization was not performed. However, normalization reduced test–retest stability with lower numbers of TMS pulses, namely with numbers below 21 (Figure 4B), which is the number of pulses currently recommended for accurate estimation of mean MEP peak to peak amplitude (31).

To validate the results obtained with MEPs collected in the right hand (i.e., when stimulating the left M1), a confirmatory analysis was applied to MEPs recorded from the left hand (i.e., resulting from right M1 stimulation), that were also collected in 30 individuals from the overall sample (50.0% female, 38.1 ± 14.5 years old, 6.7% left-handed). In data from these individuals, variability analysis for right hand MEPs resulted in similar CVs and ICCs as those obtained for the full sample (Figures 4C,D). In data collected from the left hand, non-normalized MEPs had lower between-subject variability and higher test–retest stability than that observed for the right hand (left M1). However, similarly to that found for the right hand, internal reference normalization using the largest MEPs significantly reduced between subject variability, to levels similar to those of nMEP amplitude in the right hand, even when only the initial MEPs were considered (Figure 4E). The impact of normalization on test–retest stability was again negligible, except when using only fewer MEPs, when normalization reduced temporal stability (Figure 4F).

## 4 Discussion

Here we described that internal, but not external, reference normalization using large references reduced between-subject variability of MEP amplitude, while having a small impact on test–retest stability. In the absence of normalization, between-subject variability and test–retest stability for MEPs collected in both hands fell within values reported previously for MEPs collected in the FDI (4, 41–43). Overall, external reference normalization methods did not reduce between-subject variability. In fact, in most instances, the amplitude of MEPs thus normalized had higher CVs, particularly when the largest MVIC was not used to calculate the normalization factor. However, internal reference normalization resulted in a clear reduction of between-subject variability in MEP amplitude, particularly when the largest, rather than the smallest, MEPs were used as normalization factor. With regards to the impact of normalization on MEP amplitude stability, normalization with the largest references had only a slight effect on test–retest stability, with a trend toward a slight increase of variability with use of external references and small improvement when internal references were used. However, normalization using the smaller reference signals decreased test–retest stability considerably, both for internal and external reference normalization. Our results imply that the largest MEPs may be representative of each individual’s cortical excitability during the TMS assessment, while MVICs and the smallest MEPs are less representative of an individual’s motor cortical physiology. Normalization to ABSnorm_large_ and P2Pnorm_large_ should thus be considered as candidates for reducing MEP amplitude variability in future studies.

Several analyses were supportive of the robustness of internal reference normalization using large MEPs. A strategy to reduce within-individual variability through removal of MEP outliers had the unexpected effect of increasing between-subject variability, and was indifferent relative to test–retest stability, in the absence of normalization. However, normalization decreased between-subject variability of nMEPs, to levels even lower to those found prior to outlier removal, but reduced temporal stability, demonstrating the value of normalization but also the importance of conserving outliers in analysis. Furthermore, calculations of CVs and ICCs based on increasing numbers of collected MEPs supported that the effects of normalization on reduction of between-subject variability was conserved even with as few as 10 MEPs collected per individual. However, given the reduction of test–retest stability when less than 21 MEPs were used to calculate mean MEP amplitude, considered by others (31) to be the number needed to obtain a reliable estimate of MEP amplitude, leads to our support of this as the minimal number of MEPs needed.

Interestingly, when normalization was not performed, MEPs collected in the left hand (right M1) had less between-subject variability and higher test–retest stability than MEPs collected from the right hand (left M1). This finding was still observed when limiting the analysis to subjects who performed sessions on both right and left hand. Such difference may result from our methods, with collection of MVICs in the right but not left hand, prior to eliciting MEPs. In fact, exercise has been shown to modulate MEP amplitude (44–47). This effect is dependent on exercise type (48), level of fitness (49) or presence of medical disorders (5, 50–52). Thus, the impact of MVIC collection on the measurement of MEP peak-to-peak amplitude should be taken into consideration in future studies. Nevertheless, it is noteworthy that upon internal reference normalization using large MEPs, nMEP CVs were comparable to those obtained in the left hand, further supporting the robustness of this approach, as well as its replicability. The use of internal normalization, combined with emerging MEP detection and quantization methods, could enhance nMEP replicability and further reduce variability. These methods employ advanced signal processing techniques that enable the detection of subthreshold responses. They have been demonstrated to improve the TMS-EMG signal-to-noise ratio by a factor of 5 (53).

As mentioned above, in most instances external reference normalization increased, rather than reduced, between-subject variability, particularly when the largest MVIC was not included in calculation of the normalization factor. These results indicate that participants do not perform equally across MVIC trials, and indeed only 10 of 47 subjects showed maximal muscle activation in the first MVIC trial (data not shown), emphasizing the need to repeat MVIC trials several times in order to achieve a reliable measure of maximal voluntary muscle activation (54). This conclusion is further supported by our results showing that normalization to the smallest MVICs reduced test–retest stability. Nevertheless, even in the best scenario, with the largest MVIC used for normalization, only negligible impact on between-subject variability was obtained when using any of the external reference normalization methods. Although others (55) have shown that there is a positive correlation between MVICs and MEPs collected from the tibialis anterior muscle, our study indicates an inconsistent relationship between the amplitudes of MVICs and MEPs. It is possible that the action chosen here to elicit MVICs did not produce maximum activation levels of the target muscles stimulated by TMS. Different actions or instructions might yield a better normalization profile for MVICs when used as external references. Nevertheless, using large MEPs as internal references seems to be a valid approach that is, at the very least, less sensitive to specificities in the collection of an external reference.

As previously stated, use of the smallest external references was associated with reduction of temporal stability, which may result from the need to repeat MVIC trials to achieve a reliable measure of maximal voluntary muscle activation. However, the use of the smallest MEPs as internal references also resulted in significant losses of temporal stability, as well as being less effective in the reduction of between-subject variability. The smallest MEPs are bound by the minimum amplitude required to be considered a MEP (50 μV), which may limit its ability to represent individual motor cortical excitability and serve as an effective normalization factor. In addition, small MEPs may be more susceptible to coil positioning variability, as pulses applied away from the hotspot could result in a substantial decrease in MEP amplitude. Thus, in addition to being of less use as normalization factors, their representation of pulse-specific factors, rather than factors specific for the session and/or the individual, may lead to a loss of temporal stability of the nMEP when they are used as normalization factors.

Our results should be interpreted considering potential limitations. First, since in internal reference normalization MEPs are not included in the calculation of per subject mean nMEP amplitude, the comparison between normalization strategies could have favored methods in which a larger number of MEPs are excluded. However, when the MEPs used to calculate internal normalization factors were removed in all normalization approaches, results were overlapping. Second, creating a distribution of CVs through bootstrapping could be constrained by the fact that only a small number of MEPs per subject may be available after the removal of invalid MEPs. However, sensitivity analyses limiting computations to subjects for whom at least 37 MEPs were available did not impact the results, thus supporting robustness of the bootstrapping analysis of CVs. Furthermore, the reduction of MEP amplitude between-subject variability resulting from use of internal normalization methods may be due to removal of true physiological variability, impairing capability to discriminate between populations using nMEPs (23). Future work comparing distinct populations should address this possibility.

While external references used in this work are commonly employed in EMG, they were not specifically designed for MEP normalization. These external references were chosen since we considered them a practical choice for signal normalization given that several research groups use MVICs in TMS-EMG studies to determine the active motor threshold. Notwithstanding, another potential limitation is that more complex normalization references, such as maximum M-wave, were not tested. The M-wave is the EMG signal detected as a result of transcutaneous electrical stimulation of peripheral motor nerves at a point proximal to the target muscle. Some authors have used this signal to normalize MEP amplitude measurements (4, 56, 57) and it may provide a reliable alternative for external reference normalization (23, 58). Further research should compare M-wave to internal reference normalization and the impact in discrimination between different populations.

In conclusion, here we tested the effect of different normalization procedures on between-subject variability and within-subject stability of MEP amplitude. We found that external references do not reduce between-subject variability, while internal reference normalization considerably decreases between-subject variability of MEP amplitude. Importantly, normalization to largest internal or external references have minimal impact on test–retest stability, while use of small references impairs within-subject stability. The resulting optimal normalization procedure among those tested, namely the use of large internal references was robust and replicable. We thus suggest that normalization to ABSnorm_large_ or P2Pnorm_large_ using up to three references is a viable approach to normalize MEPs, and propose that this method be tested in further research to assess its use to address clinically relevant questions.

## Data availability statement

The data supporting the conclusions of this article are not readily available but may be accessed upon reasonable request to the corresponding author.

## Ethics statement

The study was conducted in accordance with the Declaration of Helsinki and was approved by the Champalimaud Foundation Ethics Committee. Written informed consent was obtained from all participants.

## Author contributions

FFV: Writing – original draft, Writing – review & editing. GC: Writing – original draft, Writing – review & editing. DRS: Writing – review & editing. CS: Writing – review & editing. PP: Writing – review & editing. AS: Writing – review & editing. FC: Writing – review & editing. AJO-M: Writing – original draft, Writing – review & editing.
