# Supplementary material for: Reducing motor evoked potential amplitude variability through normalization
Source: Front Psychiatry. 2024 Jan 31;15:1279072. doi: 10.3389/fpsyt.2024.1279072 (PMC10864444; doi:10.3389/fpsyt.2024.1279072)
Supplement: Supplementary file 1 [file Data_Sheet_1.docx]

Supplementary Material

# In-house electromyography acquisition system validation

The following protocol was designed to assess the agreement between our in-house electromyography (EMG) acquisition system and a commonly used commercial EMG system. Hence, we recruited an independent cohort of 10 participants (50% female, 28 ± 9 years old) and followed similar technical and acquisition procedures as described in the main manuscript (please see Methods section for further details). In this protocol we have asked the participants to perform five maximum voluntary isometric contractions (MVICs) by pushing their index finger laterally, against the researchers hand, while their forearm was supported, to minimize signal contamination from other muscle sources. Each MVIC had a duration of approximately 3 s with 1 min of rest between them. Then, 40 transcranial magnetic stimulation single pulses were applied over participants’ left motor cortex.

The in-house EMG acquisition system was developed by the Scientific Hardware Development Platform at Champalimaud Foundation, which provides electronic and mechanical support to internal research (<https://www.cf-hw.org/>). This system incorporates five independent EMG channels in addition to one channel which receives direct input from the TMS machine to signal pulse delivery. The EMG acquisition parameters were based on international guidelines for collection of TMS-EMG data (Groppa et al. 2012). Data streaming was done through Bluetooth, integrated using a custom made BONSAI script (Lopes et al. 2015) and saved onto binary (.bin) files. Signal collected using the in-house EMG acquisition system were tested against the BIOPAC MP36 acquisition system (BIOPAC^®^ Systems, Inc.), a commercial solution which features built-in universal amplifiers capable of recording a wide range of physiological signals such as electrocardiogram, electroencephalogram and EMG. A suitable profile for TMS-EMG data acquisition was selected in BIPOAC. Please see a summary of the acquisition parameters for both systems in Table S1. Resulting data from BIOPAC was sent to BIOPAC Student Lab software and saved into MATLAB files (.mat). For simultaneous EMG acquisition, both systems were connected to each electrode sensor using a splitter cable (Figure S1).

Data was imported into Python3 and time-series were aligned using cross-correlation to estimate the relative time-shift between both systems. Then, data was segmented into epochs containing single MVICs and motor evoked potentials (MEPs), excluding epochs where MEP amplitude was smaller than 50μV. Finally, each epoch was linearly detrended. This procedure was performed to account for potential differences in DC amplifier offset and for potential differences in the TMS pulse artifact profile in both systems. Since the goal of this experiment was to compare the agreement between the in-house acquisition system and the commercial EMG system, no further data processing was performed.

The agreement between both systems was assessed using two strategies of wave-similarity analysis: the **linear fit method** (LFM) and the **integrated pointwise indices** (IPI) (Iosa et al. 2014; Pini, Markström, and Schelin 2022). Instead of relying on the extraction of discrete measures, such as amplitude and activation time, these methods allow to determine the agreement of the whole signal, providing a more complete comparison of the systems. Linear fit method is performed by plotting a test signal $S_{test}$ against a reference signal $S_{ref}$, characterizing a group of points in a cartesian coordinate system (($S_{ref}\left( i \right), S_{test}(i)$) for $i = 1, ... , N$ where $N$ represents the number of samples). Then, a linear regression is applied to this group of points defining:

| $\hat{Y}=a . S_{ref}+b$ | (S1) |
| --- | --- |

where $\hat{Y}$ represents the best approximation of $S_{test}$ values resulting from linear transformation of $S_{ref}$ values; $a$ represents a scaling factor, i.e. a factor by which $S_{ref}$ should be multiplied to match $S_{test}$; and $b$ which represents the scalar offset.

The LFM relies on the interpretation of the coefficient of determination ($R^{2}$) and the linear regression coefficients ($a$ and $b$), calculated by the following formulas:

| $a= \frac{\sum_{i=1}^{N} (S_{ref}\left( i \right)- \bar{S_{ref}}) . (S_{test}\left( i \right)-\bar{S_{test}})}{\sum_{i=1}^{N} {(S_{ref}\left( i \right)- \bar{S_{ref}})}^{2}}$  $b= \bar{S_{test}}-a . \bar{S_{ref}}$  $R^{2}= \frac{\sum_{i=1}^{N} (a . S_{ref}\left( i \right)+b-\bar{S_{test}})}{\sum_{i=1}^{N} {(S_{test}\left( i \right)-\bar{S_{test}})}^{2}}$ | (S2) |
| --- | --- |

From equations S2 (adapted from Iosa 2018) we can estimate the optimal values for $a$, $b$ and $R^{2}$. If $S_{test}\simeq S_{ref}$ , $a\to1$, $b\to0$ and $R^{2}\to1$. Here, MVIC and MEP epochs collected using the in-house EMG acquisition system and BIOPAC MP36 were considered test and reference signals, respectively. Then, as LFM coefficients are obtained for each epoch, the 95% confidence interval (CI) of LFM coefficients were calculated considering all epochs together as well separately for MEP and MVIC epoch, representing the agreement between both systems. Additionally, each epoch was used to calculate an **integrated pointwise index** (${IPI}_{icc}$ - Pini, Markström, and Schelin 2022). This technique consists in an extension of common reliability measures for univariate analysis, integrated over each data point of the EMG trace:

| ${IPI}_{icc}=\frac{\sum_{i=1}^{N} ICC(i)}{N-1}$ | (S3) |
| --- | --- |

Intraclass correlation coefficient for absolute agreement (ICC) was used to assess reliability. ICC for absolute agreement can be calculated using variance components of a two-way mixed effect model without interactions. As described by Pini et al., the total sum of squares of each data point i can be divided into three factors: individual (${SS}_{ind}(i)$), repetition (${SS}_{rep}(i)$) and residual (${SS}_{res}(i)$) sum of squares. Dividing each sum of squares by its corresponding degree of freedom we obtain the mean sum of squares for each factor: ${MS}_{ind}(i)$, ${MS}_{rep}(i)$ and ${MS}_{res}(i)$, respectively. ${ICC}_{aa}(i)$calculation for each data point is computed by applying equation S4:

| ${ICC}_{aa}(i)=\frac{{MS}_{ind}\left( i \right)- {MS}_{res}(i)}{{MS}_{ind}\left( i \right)+ {MS}_{res}\left( i \right)+ \frac{2}{N}\left[ {MS}_{rep}\left( i \right)+{MS}_{res}(i) \right]}$ | (S4) |
| --- | --- |

Mean ${IPI}_{icc}$ were calculated for MEP and MVIC epochs separately.

LFM and IPI analysis both reveal high agreement between the in-house and commercial acquisition systems. Please see table S2 for reliability scores of wave-similarity analyses. The LFM coefficients were close to reference values ($a\to1$, $b\to0$ and $R^{2}\to1$) and ${IPI}_{icc}$ results were higher than 0.90, indicating that excellent agreement was found between the in-house and commercial EMG systems (Koo and Li 2016). A confirmatory analysis using ICCs of mean MEP and MVIC amplitudes also revealed excellent agreement between both systems with ICC=1.00, 95%CI [1.00, 1.00] and ICC=0.96, 95%CI [0.87, 0.99] for mean MEPs and MVICs, respectively. These results support the use of our in-house built EMG acquisition system for extracting TMS-EMG data.

Supplementary material is not typeset so please ensure that all information is clearly presented, the appropriate caption is included in the file and not in the manuscript, and that the style conforms to the rest of the article.

# Supplementary Figures and Tables

## Supplementary Figures

### Supplementary Figure 1


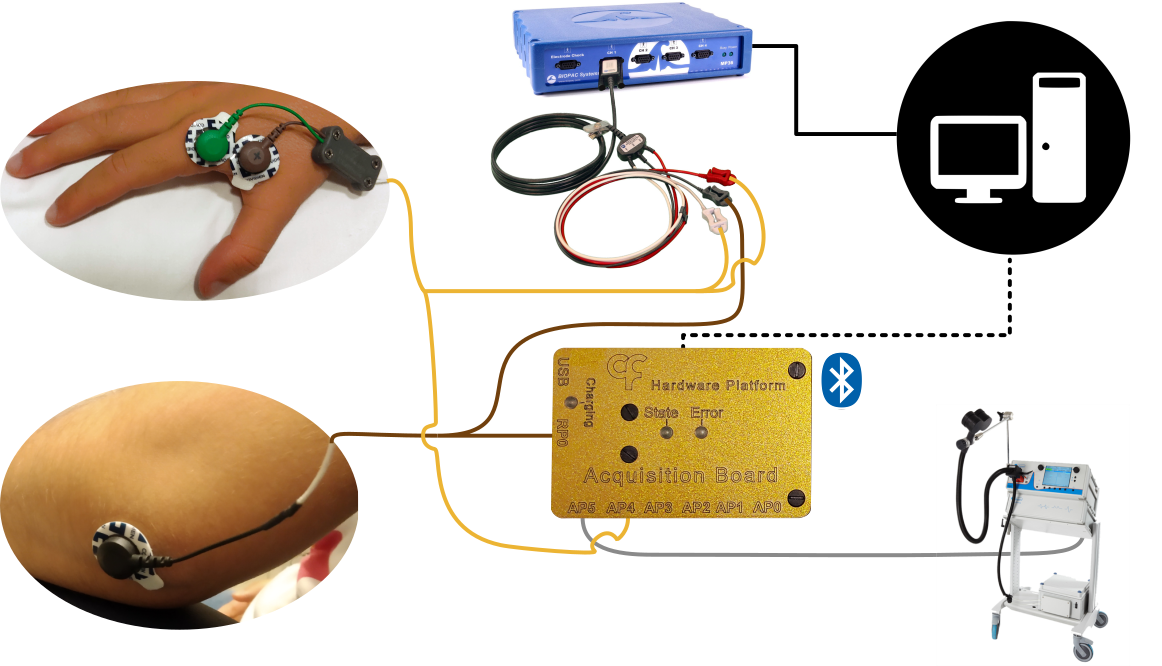


**Supplementary Figure 1 - In-house EMG acquisition system validation setup**. Simultaneous EMG acquisition was achieved by connecting both systems to electrode sensors with a splitter cable.

### Supplementary Figure 2


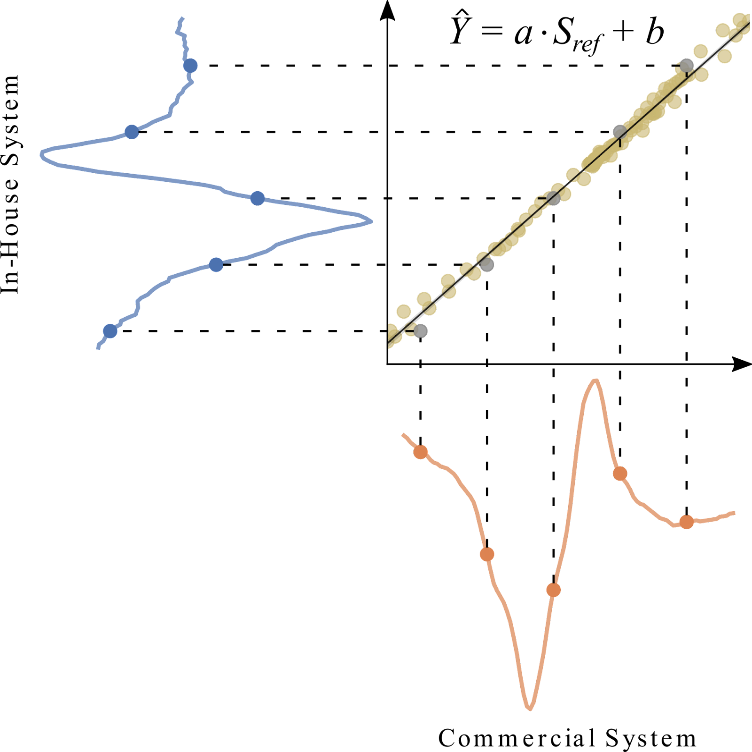


**Supplementary Figure 2 - Illustration of the Linear Fit Method** applied to an example motor evoked potential.

## Supplementary Tables

### Supplementary Table 1

| **Table S1. Summary of EMG acquisition system parameters.**  Abbreviations: ADC - Analog to Digital Converter | | |
| --- | --- | --- |
|  | **In-house EMG acquisition system** | **Commercial System** |
| Gain | 206 | 200 |
| Sampling Frequency | 5000 Hz | 5000 Hz |
| Hardware Passband | 5 Hz - 1000 Hz | 5 Hz - 20 kHz |
| ADC Sampling resolution | 16 bits | 24 bits |
| Max Input voltage range | ± 7.3 mV | ± 2 V |

### Supplementary Table 2

| **Table S2. Wave-similarity analyses results**. Linear fit method coefficients are represented by the 95% confidence interval. Integrated pointwise indices using the intra-class correlation coefficient for absolute agreement as reliability measure showed excellent reliability (larger than 0.90). Abbreviations: MEP – Motor Evoked Potential; MVIC – Maximum Voluntary Isometric Contraction. | | | | |
| --- | --- | --- | --- | --- |
|  | | **Both** | **MEP epochs** | **MVIC epochs** |
| Linear Fit Method | $a$ | [0.924, 0.934] | [0.929, 0.949] | [0.878, 0.901] |
|  | $b$ | [-0.471, -0.214] | [-0.539, -0.246] | [-0.045, 0.036] |
|  | $R^{2}$ | [0.953, 0.964] | [0.962, 0.972] | [0.882, 0.929] |
| Integrated Pointwise Index | ${IPI}_{icc}$ |  | 0.951 | 0.933 |

# References

Groppa, S., A. Oliviero, A. Eisen, A. Quartarone, L.G. Cohen, V. Mall, A. Kaelin-Lang, et al. 2012. ‘A Practical Guide to Diagnostic Transcranial Magnetic Stimulation: Report of an IFCN Committee’. *Clinical Neurophysiology* 123 (5): 858–82. https://doi.org/10.1016/j.clinph.2012.01.010.

Iosa, M., A. Cereatti, A. Merlo, I. Campanini, S. Paolucci, and A. Cappozzo. 2014. ‘Assessment of Waveform Similarity in Clinical Gait Data: The Linear Fit Method’. *BioMed Research International* 2014: 1–7. https://doi.org/10.1155/2014/214156.

Koo, Terry K., and Mae Y. Li. 2016. ‘A Guideline of Selecting and Reporting Intraclass Correlation Coefficients for Reliability Research’. *Journal of Chiropractic Medicine* 15 (2): 155–63. https://doi.org/10.1016/j.jcm.2016.02.012.

Lopes, Gonçalo, NiccolÃ^2^ Bonacchi, JoÃ£o FrazÃ£o, Joana P. Neto, Bassam V. Atallah, Sofia Soares, LuÃ­s Moreira, et al. 2015. ‘Bonsai: An Event-Based Framework for Processing and Controlling Data Streams’. *Frontiers in Neuroinformatics* 9 (April). https://doi.org/10.3389/fninf.2015.00007.

Pini, Alessia, Jonas L Markström, and Lina Schelin. 2022. ‘Test–Retest Reliability Measures for Curve Data: An Overview with Recommendations and Supplementary Code’. *Sports Biomechanics* 21 (2): 179–200. https://doi.org/10.1080/14763141.2019.1655089.
